# Clarifying CLARITY: Quantitative Optimization of the Diffusion Based Delipidation Protocol for Genetically Labeled Tissue

**DOI:** 10.3389/fnins.2016.00179

**Published:** 2016-04-25

**Authors:** Chiara Magliaro, Alejandro L. Callara, Giorgio Mattei, Marco Morcinelli, Cristina Viaggi, Francesca Vaglini, Arti Ahluwalia

**Affiliations:** ^1^Research Center “E. Piaggio”, University of PisaPisa, Italy; ^2^Department of Translational Research and New Technologies in Medicine and Surgery, University of PisaPisa, Italy

**Keywords:** CLARITY, image processing, quantitative protocol optimization, mouse brain slices, GFP

## Abstract

Tissue clarification has been recently proposed to allow deep tissue imaging without light scattering. The clarification parameters are somewhat arbitrary and dependent on tissue type, source and dimension: every laboratory has its own protocol, but a quantitative approach to determine the optimum clearing time is still lacking. Since the use of transgenic mouse lines that express fluorescent proteins to visualize specific cell populations is widespread, a quantitative approach to determine the optimum clearing time for genetically labeled neurons from thick murine brain slices using CLARITY2 is described. In particular, as the main objective of the delipidation treatment is to clarify tissues, while limiting loss of fluorescent signal, the “goodness” of clarification was evaluated by considering the bulk tissue clarification index (BTCi) and the fraction of the fluorescent marker retained in the slice as easily quantifiable macroscale parameters. Here we describe the approach, illustrating an example of how it can be used to determine the optimum clearing time for 1 mm-thick cerebellar slice from transgenic L7GFP mice, in which Purkinje neurons express the GFP (green fluorescent protein) tag. To validate the method, we evaluated confocal stacks of our samples using standard image processing indices (i.e., the mean pixel intensity of neurons and the contrast-to-noise ratio) as figures of merit for image quality. The results show that detergent-based delipidation for more than 5 days does not increase tissue clarity but the fraction of GFP in the tissue continues to diminish. The optimum clearing time for 1 mm-thick slices was thus identified as 5 days, which is the best compromise between the increase in light penetration depth due to removal of lipids and a decrease in fluorescent signal as a consequence of protein loss: further clearing does not improve tissue transparency, but only leads to more protein removal or degradation. The rigorous quantitative approach described can be generalized to any clarification method to identify the moment when the clearing process should be terminated to avoid useless protein loss.

## Introduction

One of the challenges of modern neuroscience is to map the architecture of neural circuits in the mammalian brain, in order to delineate the so-called “Connectome” (Van Essen and Ugurbil, 2012), tracing the information pathways through axons and dendrites of neurons in their native three-dimensional (3D) arrangement.

The main obstacle for this kind of study is the presence of lipids, which cause light scattering, limit the depth of light penetration, and constitute an antibody-impermeable barrier. Even using two-photon microscopy, it is impossible to penetrate brain samples more than a few hundred microns (Oheim et al., 2001), which is insufficient for reconstructing large brain projections or complete neural populations (Chung and Deisseroth, 2013).

To overcome these limits, a number of optical clearing or delipidation approaches have been developed to render the whole brain transparent so that it can be analyzed without sectioning (Hama et al., 2011; Ertürk et al., 2012; Ke et al., 2013; Kuwajima et al., 2013; Richardson and Lichtman, 2015). Among these, the CLARITY method, pioneered and disseminated through forums and Wiki pages by Deisseroth's group (i.e., http://forum.claritytechniques.org/), has captured the imagination of many researchers and is currently discussed and debated widely (Chung and Deisseroth, 2013; Chung et al., 2013; Tomer et al., 2014). To date about 20 new papers on CLARITY and its variations have been published and a number of them are dedicated to the optimization or simplification of the experimental set up (Lee et al., 2014; Epp et al., 2015; Esposito and Nikitichev, 2015; Zheng and Rinaman, 2015). In fact, despite the plethora of virtual discussion groups, the method remains substantially heuristic due to the large number of steps involved and the ensemble of variables which contribute to the tissue delipidation process. For instance, tissue clearing is evaluated by visible inspection and is thus prone to observer bias. Furthermore, the mechanisms of tissue fixing and clarification remain elusive, making it almost impossible to standardize CLARITY for rigorous quantitative studies.

After Chung et al.'s seminal report (Chung et al., 2013), a simplified diffusion based method, CLARITY2, was proposed by Poguzhelskaya et al. (2014) to clarify 1–1.5 mm thick slices. CLARITY2 does not necessitate the use of the electrophoretic chamber—probably the most time consuming and difficult step of the whole procedure. The passive clarity technique (PACT) is very similar to CLARITY2 (Yang et al., 2014). Both approaches can be very useful when it is not necessary to achieve the full potential of CLARITY to delipidate an intact brain, focusing the study only on a brain sub-region or on a specific neuronal population (i.e., Purkinje cells in the cerebellar layers). However, despite their excellent contribution to brain imaging, even passive clearing methods combine a large number of variables (i.e., slice thickness, reagent concentrations, clearing times) leaving much to trial and error.

Like CLARITY, CLARITY2 involves protein and structural fixation through the use of formaldehyde and an acrylamide based gel, followed by solubilisation of lipids using sodium dodecyl sulfate (SDS) in the so-called clearing solution. The removal of tissue lipids reduces tissue opacity, but is inevitably accompanied by a non-specific loss of inter- and extra-cellular components. Protein loss is in fact unavoidable during tissue clarification, for example Chung et al.'s original paper (Chung et al., 2013) reports an 8% decrease in protein content, albeit some scientists on the CLARITY forum claim complete loss of GFP signal (http://forum.claritytechniques.org/discussion/32/loss-of-gfp-signal). Although, light can propagate further into a highly transparent clarified tissue, there is less probability of exciting fluorescence in such samples; hence the effective measured signal is reduced.

Mouse lines are successfully and widely used to visualize specific cell populations in the brain by the transgenic expression of fluorescent proteins (Abe and Fujimori, 2013). In genetically labeled tissue, clarification for imaging the 3D cellular architecture can be regarded as a trade-off between the increase in light penetration depth due to delipidation and a decrease in phenotypic emission signal as a consequence of protein loss through solubilisation or degradation.

To determine the best compromise between transparency and fluorescent signal, an experimental method was developed for optimization and standardization of the CLARITY2 protocol. In particular, keeping all reagent concentrations as established by Chung et al. (2013) constant, we define an approach to macroscopically assess the delipidation efficacy as a function of clearing time and describe an example of how this method can be used to identify the best clearing time for 1 mm-thick cerebellar slices obtained from L7GFP mice, in which GFP (green fluorescent protein) expression is driven by the Pcp-2 promoter and is specific for Purkinje cells (PCs) in the cerebellar layers (Zhang et al., 2001). In parallel, to double-check our experimental method, the results were compared with those obtained with indexes usually used in image processing.

## Materials and methods

### Tissue preparation

L7GFP and wild-type (WT) mice were obtained from the Department of Translational Research, New Technologies in Medicine and Surgery of the University of Pisa (Italy). Mice were used to perform the experiments, which were conducted in conformity with the European Communities Council Directive of 24 November 1986 (86/609/EEC and 2010/63/UE) and in agreement with the Italian DM26/14. Experiments were approved by the Italian Ministry of Health and Ethical Committee of the University of Pisa. Adult mice were anesthetized with a lethal dose of 7% chloral hydrate and then perfused at a slow flow rate (about 2 min for 20 mL of solution) with 20 mL of ice cold Phosphate Buffered Saline (PBS 1X, Sigma-Aldrich, Milan, Italy) and then 20 mL of ice cold hydrogel solution, containing 4% acrylamide, 0.05% bis-acrylamide (Biorad Lab Inc., California, USA), 4% formaldehyde (PFA, Sigma-Aldrich) and 0.25% VA-044 thermally triggered initiator (Wako Chemicals, Neuss, Germany) at 4°C, as described in Chung et al. (2013). The brain was immediately extracted and submerged in 20 mL hydrogel solution for 3 days at 4°C in a 50 mL Falcon tube (covered with aluminum foil to protect samples from direct light exposure) to allow gel diffusion into the tissue. Then the cap was substituted with a modified one with a small hole to which a short piece of silicone tube with an on-off valve was hot-glued. A vacuum was applied to the tube for 10 min, after which the valve was closed to enable hydrogel formation in the absence of air (the presence of oxygen impedes gelation of the acrylamide gel). Polymerization of the biomolecule-conjugated monomers in the hydrogel mesh was thermally initiated by incubating the infused tissue overnight at 37°C. At this point, the mouse brain was isolated by carefully removing the surrounding excess hydrogel, and vertically cut along to the coronal plane with a scalpel to obtain the portion containing the cerebellum. The latter was then cut into 1 mm-thick coronal slices using a Leica VT1200S vibratome (Leica Microsystems, Nussloch, Germany) with a stainless steel razor blade (Gillette, Milan, Italy). The cut settings were: blade angle, 18°; sectioning speed, 0.2 mm/s; and oscillating amplitude, 1.5 mm (Mattei et al., 2015). Each hydrogel-embedded slice was placed in a 50 mL Falcon tube at 37°C with 20 mL of CLARITY clearing solution, composed of 200 mM Boric Acid (Farmitalia Carlo Erba spa, Italy) and 4% Sodium Dodecyl Sulphate (SDS, Sigma-Aldrich) (Chung et al., 2013). The pH was adjusted to 8.5 by adding 1 M NaOH dropwise. Cerebellar slices embedded in the hydrogel and immersed in 20 mL of 1X PBS solution were used as controls. Clearing solutions and PBS in the controls were changed at 3, 5, and 7 days.

### Quantitative evaluation of clarification

At each time point investigated (i.e., day 0−3−5−7−10), the “goodness” of clarification for both the treated tissue slices and controls was evaluated using two macroscopic approaches: (i) a quantification of tissue clarification through image analysis, and (ii) an evaluation of the fraction of the protein-of-interest lost in the clearing solution.

#### Bulk tissue clarification index

**Figure 1:**
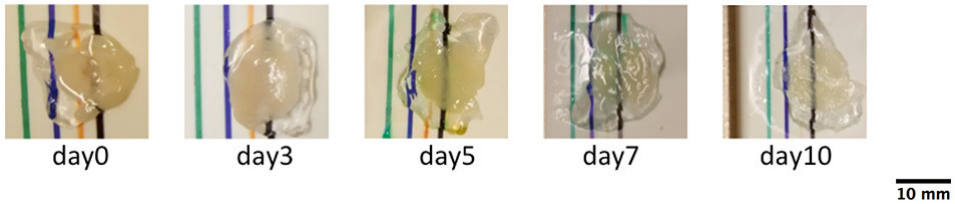
**Photographs of cerebellar slices at different clearing times**. The images were used to calculate BTCi through Equation (1), comparing the intensity of the black line below the slice and in the region without the slice. (pixel size: 0.125 mm).

Tissue slices were placed on a white plastic support marked with a black line and photographed using a Nikon D5100 reflex camera (Figure 1). After converting the images into 8-bit grayscale, the clarification was evaluated defining a Bulk Tissue Clarification index (BTCi) as:

(1) $$\begin{array}{l} BTCi=\frac{{255-I}_{1}}{255-I_{s}} \end{array}$$

where *I*_1_ and *I*_*s*_ are the mean pixel intensities of the black line traced on the support respectively with and without the tissue on it. Averaging the pixel intensities reduces local variations, while the term *I*_*s*_ in Equation 1 serves as an internal normalization which eliminates variations due to any differences in environmental light conditions between images. The index so-defined ranges from 0 (i.e., totally opaque white slice) to 1 (i.e., totally transparent slice).

#### Quantification of the fluorescent protein loss

In principle, there are two sources of tissue protein loss: protein denaturation due to the clearing solution and protein release in the clearing solution. Since the clearing solution (pH 8.5 containing SDS) is not likely to denature biological fluorophores (Saeed and Ashraf, 2009), the loss of fluorescent protein from the slice can be assumed equal to that released in the clearing solution. In this study, to quantify GFP loss, at each time point, 200 μL samples of the clearing solution were analyzed in triplicate with a plate reader (FLUOstar Omega, BMG Labtech, Ortenberg, Germany, Ex: 485 nm and Em: 544 nm). Fluorescence was read against a blank of fresh clearing solution, keeping the spectrofluorimeter settings (e.g., gain, number of flashes per well) constant over measurements to enable meaningful quantitative analyses of fluorescent protein release over time. Since the clearing solution was completely refreshed at day 3, 5, and 7, for each brain slice the fluorescence data obtained at different time points were summed and expressed as cumulative fluorescence until day 10. To exclude fluorescent contributions due to tissue degradation or autofluorescence, no-labeled tissue slices were also clarified and clearing solution samples were taken at the same time points (i.e., day 3–5–7–10) analyzed with the same plate-reader settings.

### Evaluation of image quality using standard indices

Image stacks of slices treated with clearing solution and controls were mounted on a glass slide with FocusClear™ (Celexplorer Labs Co., Hsinchu, Taiwan) and then acquired with a confocal microscope (Nikon A1) at different time points. In particular, a 200 μm z-stack with a step size of 2 μm was acquired using a 10X objective with a pixel-to-micron ratio of 0.46 μm/pixel on a 512 × 512 matrix. The same confocal settings were used for all scans (i.e., 4.84 W laser power, emission and excitation wavelengths of 488 nm and 502 nm respectively). Two widely used indices for quantifying image quality were calculated:

#### (i) Mean pixel intensity

To quantify tissue clarification as function of time, the Mean Pixel Intensity (MPI) of the objects of interest (PCs) was evaluated using the method described by Gonzalez et al. (2009). An automated algorithm was developed in Matlab (The Mathworks Inc.) to estimate the MPI for each of the 100 images in the z-stack. First a global threshold with Otsu's method (Otsu, 1979) was performed for every plane image of the stack to identify the objects (i.e., the PCs). Then, the MPI of thresholded objects in each plane was calculated using:

(2) $$MPI=\frac{\sum_{i\text{=1}}^{i=M}I_{M}}{M}$$

where *M* is the number of object pixels and *I*_*M*_ their pixel intensity.

#### (ii) Contrast-to-noise ratio (CNR)

Although, the MPI is a measure of the signal, it is not directly linked to the information content of the image, which also depends on contrast between labeled neurons and the background. An alternative parameter for evaluating light scattering through the depth of the slice taking into account the image background is the Contrast-to-Noise (CNR) ratio defined as:

(3) $$\begin{array}{l} CNR\;=\frac{MPI-I_{b}}{\sqrt{\frac{\sigma_{m}+\sigma_{b}}{2}}} \end{array}$$

where *MPI* is as defined previously, *I*_*b*_ is the mean intensity of the background, σ_*m*_ is the standard deviation of the objects and σ_*b*_ the standard deviation of the background (Song et al., 2004).

Once again, the CNR was calculated using an automated routine in Matlab. For each image in the stack, the Otsu-based thresholding method described was used to identify the objects of interest and discriminate them from the background. Then, assuming σ_*m*_ = σ_*b*_, as proposed by Song and co-workers (Song et al., 2004), the CNR was calculated according to Equation (3).

### Sample evaluation and statistical analysis

Five animals were employed for the MPI and CNR analyses, using *n* = 2 slices per day for both experimental samples and controls. Thus, a total of *n* = 18 slices were employed, 2 for the time 0 analyses and 16 for the other time-points, i.e., 2 (replicates) × 4 (time points) × 2 (treatments). Each sample was imaged in 4 different regions, thus averaging 8 datasets from 2 slices per data point. Sample from different animals were pooled together as replicates for the analyses, assuming no inter-animal differences.

Six animals, three L7GFP mice and three WTs, were used for the macroscale BTCi and protein loss experiments, again pooling samples together. Here a total of *n* = 10 slices (5 controls in PBS + 5 samples in clearing solution) were used throughout the 10 days to determine average daily values of BTCi and GFP leakage. Unlike the MPI and CNR analyses, BTCi and GFP loss experiments were performed on the same slice until day 10, obtaining 5 replicates per experiment and time point investigated.

Statistical analyses of BTCi, GFP loss, MPI, and CNR data were carried out using ANOVA followed by Tukey's Multiple Comparison Test, setting significance at *p* < 0.05.

## Results

### Bulk tissue clarification index (BTCi) evaluation

Assuming no differences in the gross optical properties between cerebellum slices, data from different sections acquired at the same time points were grouped together as sample replicates to evaluate the BTCi.

**Figure 2:**
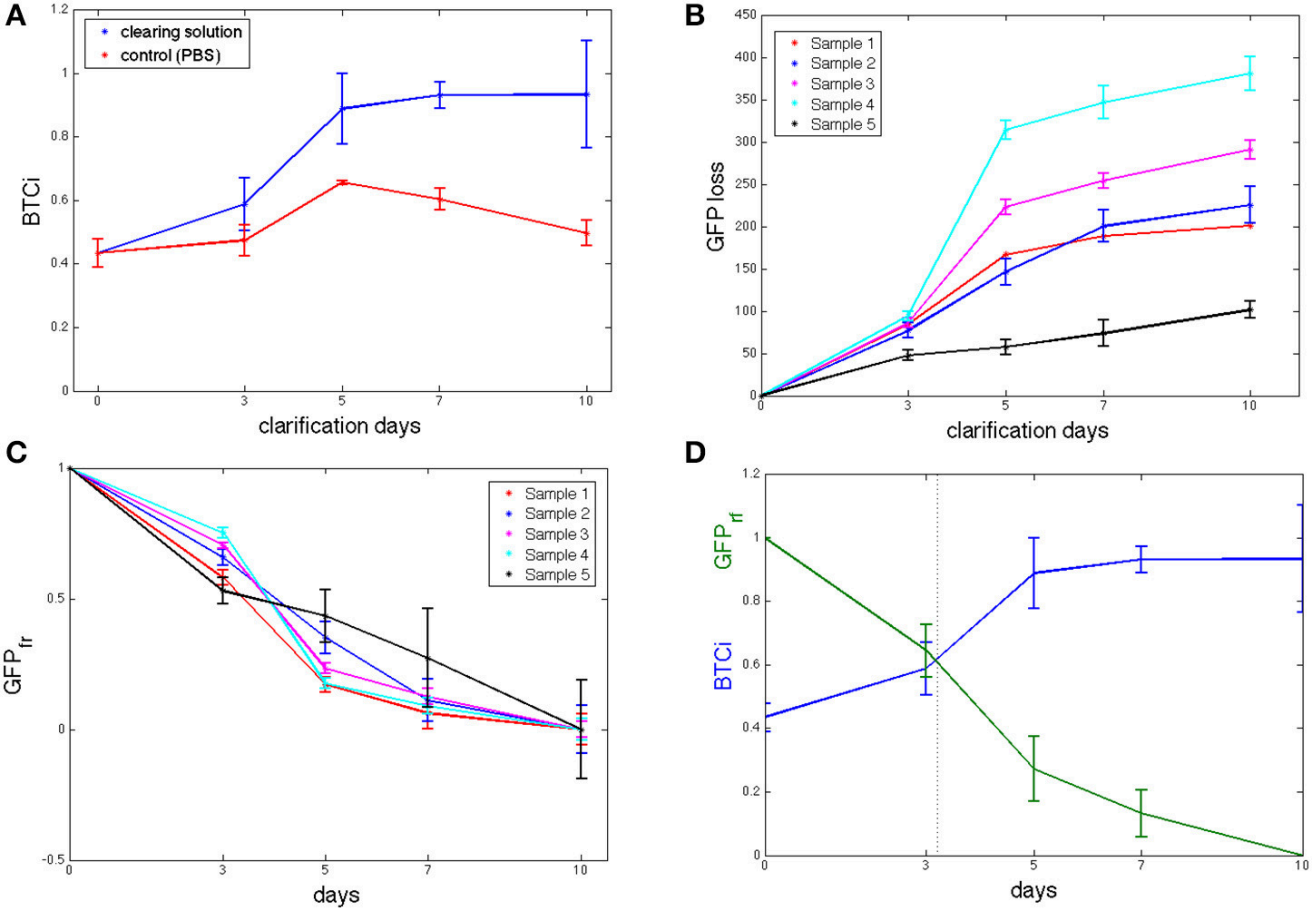
**(A)** BTCi as a function of clearing time for control cerebellar slices in PBS (*n* = 5, red) and in CLARITY clearing solution (*n* = 5, blue) slices. **(B)** Cumulative GFP measured in the clearing solution over time (*n* = 5 slices). **(C)** Fraction of GFP retained (*GFP*_*fr*_), expressed as in equation 4, showing no significant differences between slices at the same time point. **(D)** BTCi and *GFP*_*fr*_ time series obtained grouping results from the 5 different slices together, showing the relationship between the two parameters.

The initial BTCi for untreated slices (i.e., time 0 in Figure 2A) was 0.43 ± 0.04. For samples immersed in clearing solution, this index increases significantly over time (*p* > 0.05, one-way ANOVA) until it reaches a plateau at day 5 (BTCi = 0.88 ± 0.11). On the other hand, the BTCi does not change significantly over time (*p* > 0.05, one-way ANOVA) for cerebellar slices immersed in PBS (i.e., the negative control).

### Evaluation of GFP fraction lost

As fluorescence from un-labeled tissue slices was similar to that of the virgin clearing solution, we can assume that there are no fluorescent components from tissue degradation or autofluorescence of brain components, and so all the signal detected by the spectrofluorimetric analysis refers to GFP loss in the clearing solution. The cumulative GFP loss from each sample is reported in Figure 2B. Data for GFP loss in controls are not shown as the values were either negative or close to zero, indicating levels of GFP close to or below the limit of detection. As shown in the figure, the rate of GFP leaked is initially high but tends to decrease with time toward an equilibrium value, typical of passive diffusion. Although, the trend for all slices examined is similar, it is not possible to assume a slice-independency as supposed for the BTCi evaluation. Indeed, the amount of GFP leaked into the clearing solution, expressed as arbitrary fluorescence units, varies from slice to slice because of the heterogeneous distribution of PCs in the cerebellum (Figure 2B). Hence an appropriate normalization is needed to meaningfully compare results from different slices. Assuming GFP loss is a diffusive process, each sample loses the same fraction of protein at equilibrium. Since the cumulative GFP release did not change significantly between day 7 and 10 (i.e., the release of GFP appears to have reached a plateau and does not increase significantly over time), we assume that day 10 corresponds to the equilibrium state. Therefore, to normalize the loss of fluorescent protein (*FP*_*loss*_) from each slice, the cumulative fluorescence values obtained from the clearing solutions were divided by their respective values at day 10, *FP*_*loss*_(*t*_*end*_). A first one-way ANOVA analysis was performed on normalized GFP data obtained at each of the time points investigated to verify that this parameter is not slice-dependent (Figure 2C). Then, data from different slices collected at the same time point were grouped together to give the fraction of fluorescent protein retained, *FP*_*fr*_:

(4) $$\begin{array}{l} {FP}_{fr}\left(t\right)=1-\frac{{FP}_{loss}\left(t\right)}{{FP}_{loss}\left(t_{end}\right)} \end{array}$$

The fraction of fluorescent protein retained decreases continuously over time, as expected for passive diffusion (Figure 2D). The corresponding BTCi is plotted in the same graph to highlight the correlation between tissue transparency and GFP specific fluorescence of the sample.

### MPI and CNR evaluation

**Figure 3:**
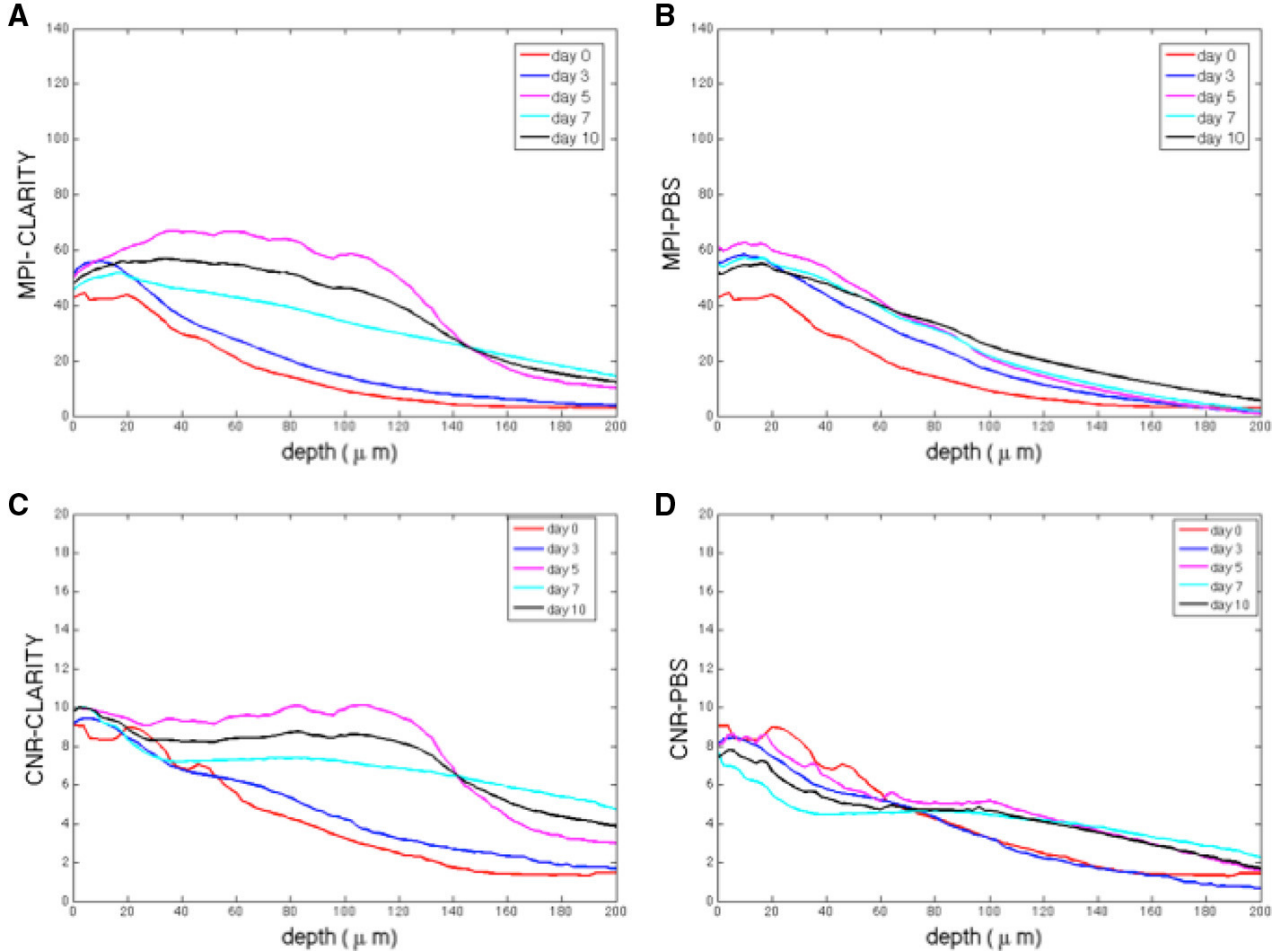
**(A)** Mean pixel intensity (MPI) as a function of stack depth for tissue slices immersed in clearing solution for different times (*n* = 2 slices per line). **(B)** MPI for controls (*n* = 2 slices per line). **(C)** CNR (contrast to noise ratio) as a function of stack depth for tissue slices immersed in clearing solution for different times (*n* = 2 slices per line). **(D)** MPI for controls (*n* = 2 slices per line). For each sample acquired, the MPI and CNR were calculated over 200 μm thick regions from 100 different images spaced 2 μm apart, i.e., a total of 100 data points).

**Figure 4:**
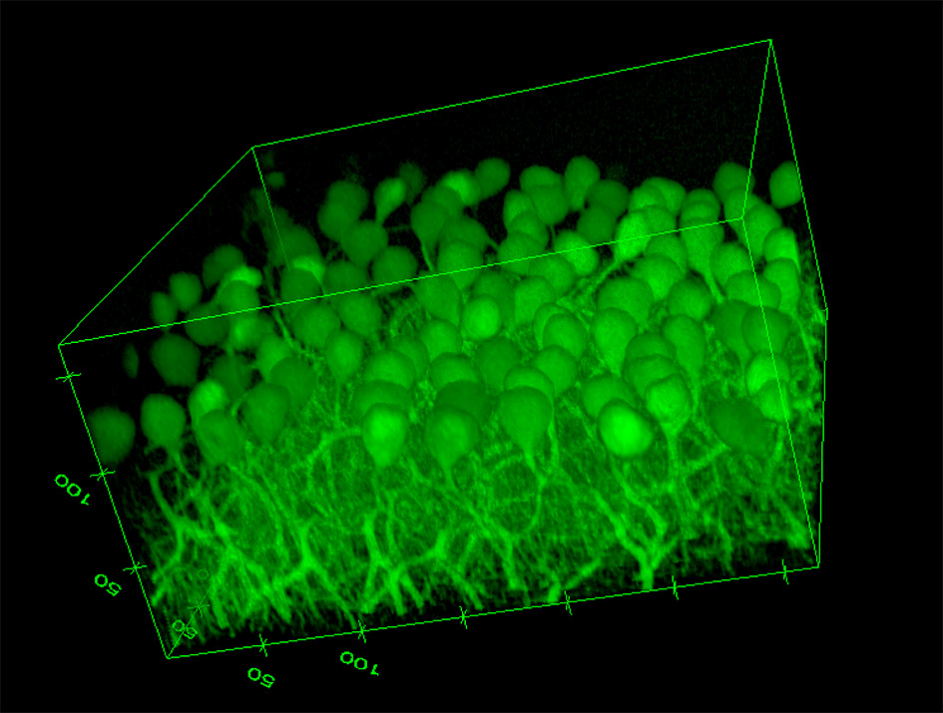
**Volume view of a confocal stack acquired at day 5 (Ex/Em: 488/502, pixel-to micron ratio: 0.62 μm, z-resolution: 1.2 μm)**. Volume dimensions (w × l × h): 317 × 317 × 172 μm (numbers in the edges of the box represent distances in microns).

The image based MPI and CNR analyses of the confocal z-stack images are reported in Figure 3. For the sake of clarity, only the mean values are shown. Although, the variations are not statistically significant due to the unavoidable intrinsic variations between slices and the heterogeneous distribution of cells in each region imaged, there is a notable difference in trends between the samples immersed in the CLARITY clearing solution and PBS. The MPI decreases with increasing depth in controls and this trend is fairly independent of the number of days the slice is immersed in PBS (Figure 3B). On the other hand, in clarified tissues the MPI decreases with depth during the first few days in clearing solution and then increases inside the slices, reaching the highest values at day 5 (Figure 3A). Similar results are obtained for the CNR (Figure 3C for the slices immersed in clearing solution and Figure 3D for the PBS-immersed ones); for a given depth in the sample the highest CNR values are found at day 5 of clarification. Figure 4 shows a volume view of a 3D reconstruction from a confocal stack acquired at day 5, while Video 1 (see Supplementary Materials) is an animation of the cerebellum slice.

## Discussion

Most investigations on the optimization of CLARITY and its variants focus on the composition of the hydrogel embedding and clearing solutions and on design of the electrophoretic chamber. Whatever the method and reagents used, the “goodness” of any clearing process is essentially the best trade-off between tissue transparency and the presence of molecules of interest to imaging. For a given clearing cocktail, the former increases with clearing time, while the latter are inevitably lost due to a shift in equilibrium between tissue bound and unbound moieties or protein degradation. The aim of this study was therefore to design a method to characterize the clearing process as a function of time and so determine the optimum clearing time for thick brain slices.

The approach described was applied to 1 mm-thick cerebellum slices of L7GFP mice, whose Purkinje neurons are fluorescent labeled, using the diffusion-based CLARITY2 method. The “goodness” of clarification was quantified by evaluating both the bulk tissue clarification index (BTCi) and the fraction of GFP lost in the clearing solution. Figure 2D summarizes the main results of the macroscale analyses: BTCi increases with clearing time, reaching a plateau after 5 days, while the fraction of GFP retained decreases rapidly. To attest the validity of the approach, brain slices were also imaged using confocal microscopy to calculate the MPI of neurons and the CNR as a function of z-stack depth. The results show that slices cleared for 5 days have the highest MPI and CNR for the widest range of depths. This trend is not observed for PBS-immersed slices.

Thus, the delipidation time can be optimized by measuring the bulk tissue clarification index and the fraction of protein-of-interest lost in clearing solution simultaneously. The optimum clearing time is when tissue clarification just reaches its maximum, as any further clearing leads to excessive and useless signal loss. Prolonging the clarification treatment does not significantly improve tissue transparency and may also be detrimental for the maintenance of the sample's architectural and biochemical features. In case of fluorescent proteins less stable than GFP and its relatives, or for non-genetically labeled tissues, the latter can be measured with different complementary techniques (i.e., the BCA (bicinchoninic acid) protein assay), assuming the all the proteins behave in the same way during the diffusion processes).

The approach proposed in this paper can be generalized and adopted for the quantification and optimization of other optical clearing methods (e.g., 3Ddisco, PACT etc.) and/or different organs, animals or sample size.

Once sufficient lipids have been removed to attenuate scattering [the main cause of tissue opacity (Jacques, 2013)] without compromising protein loss, Focus Clear or alternative solutions with a high real (i.e., *n*) and low imaginary refractive index (i.e., *jk* or attenuation coefficient) can increase tissue transparency post-clearing as recently demonstrated by Costantini et al. (2015).

## Author contributions

CM, AC, GM, FV, and AA designed the research; CM, AC, MM and CV performed the research; CM, AC and GM analyzed the data; CM, GM and AA wrote the paper. All authors read and approved the final manuscript.

## Funding

This study was supported by PRA_2016_56 of the University of Pisa.

### Conflict of interest statement

The authors declare that the research was conducted in the absence of any commercial or financial relationships that could be construed as a potential conflict of interest. The reviewer YM and handling Editor declared their shared affiliation, and the handling Editor states that the process nevertheless met the standards of a fair and objective review.
